# Pulse wave velocity and carotid atherosclerosis in White and Latino patients with hypertension

**DOI:** 10.1186/1471-2261-11-15

**Published:** 2011-04-11

**Authors:** Mori J Krantz, Carlin S Long, Patrick Hosokawa, Elhum Karimkahani, Miriam Dickinson, Raymond O Estacio, Frederick A Masoudi, Edward P Havranek

**Affiliations:** 1 Cardiology Division, Denver Health Medical Center, Denver, USA; 2 Department of Medicine, University of Colorado School of Medicine, Aurora, USA; 3 Department of Family Medicine, University of Colorado School of Medicine, Aurora, USA

**Keywords:** Pulse wave velocity, hypertension, atherosclerosis, carotid intima media thickness, Latino, inflammatory markers, augmentation index, central aortic pressure, C-reactive protein

## Abstract

**Background:**

Preventive cardiology has expanded beyond coronary heart disease towards prevention of a broader spectrum of cardiovascular diseases. Ethnic minorities are at proportionately greater risk for developing extracoronary vascular disease including heart failure and cerebrovascular disease.

**Methods:**

We performed a cross sectional study of Latino and White hypertension patients in a safety-net healthcare system. Framingham risk factors, markers of inflammation (hsCRP, LPpLA2), arterial stiffness (Pulse wave velocity, augmentation index, and central aortic pressure), and endothelial function (brachial artery flow-mediated dilatation) were measured. Univariate and multivariable associations between these parameters and an index of extracoronary atherosclerosis (carotid intima media thickness) was performed.

**Results:**

Among 177 subjects, mean age was 62 years, 67% were female, and 67% were Latino. In univariate analysis, markers associated with carotid intima media thickness (IMT) at p < 0.25 included pulse wave velocity (PWV), augmentation index (AIx), central aortic pressure (cAP), and LpPLA_2 _activity rank. However, AIx, cAP, and LpPLA2 activity were not significantly associated with carotid IMT after adjusting for Framingham risk factors (all p > .10). Only PWV retained a significant association with carotid IMT independent of the Framingham general risk profile parameters (p = .016). No statistically significant interactions between Framingham and other independent variables with ethnicity (all p > .05) were observed.

**Conclusion:**

In this safety net cohort, PWV is a potentially useful adjunctive atherosclerotic risk marker independent of traditional risk factors and irrespective of ethnicity.

## Background

An emerging concept in cardiovascular disease (CVD) prevention is a shift in focus from predicting isolated coronary events to broader prediction of any CVD event. This has led to derivation of a new Framingham CVD risk profile [1] beyond the traditional score for predicting 10-year coronary disease events [2]. To illustrate this, data from the National Health and Nutrition Examination Surveys compared the two instruments among US men and women and found that just 3% of individuals were at a high (>20%) predicted coronary disease risk, whereas 18% were at high CVD risk when CVD death, myocardial infarction, angina, stroke, transient ischemic attack, peripheral arterial disease, and heart failure were encompassed [3]. This broader focus on predicting CVD risk might especially benefit ethnic minorities given higher prevalence rates of non-coronary vascular disease [4]. Although the prevalence of coronary disease is higher for Whites versus minorities, total CVD rates are higher among minorities, largely attributable to a higher occurrence of stroke and heart failure [4].

At the same time, considerable efforts have been made to improve the discriminative capacity of risk prediction tools by adding new risk factors to the models. Markers of inflammation, particularly high-sensitivity C-reactive protein (hsCRP), and measures of vascular function have received attention. The role of emerging markers has not been well explored for prediction of CVD beyond coronary disease, and data are more limited among minority populations. For instance, just 1% of subjects in both derivation and validation cohorts of a study assessing the additional predictive value of hsCRP were Latino [5]. Although a greater prevalence of arterial stiffness has been demonstrated in African Americans [6], little is known about vascular function among Latinos, the largest and fastest growing minority population in the United States [7]. Given this background, we evaluated inflammatory and vascular function markers in a safety-net population with chronic hypertension. We hypothesized that regardless of ethnicity, pulse wave velocity (PWV) would be associated with pre-clinical carotid atherosclerosis beyond traditional risk factors.

## Methods

### Subject characteristics

The study sample consisted of 177 subjects recruited from an electronic registry of hypertension patients created as part of the Latinos Using Cardio Actions to Reduce Risk (LUCHAR) program [8]. Patients in the registry were regular patients at Denver Health, an integrated urban safety net health system [9]. Data from the registry were supplemented with chart review and patient self-report during an enrollment interview. Patients were eligible for the current study if they were ≥18 years of age, of either Latino or non-Latino White ethnicity, actively receiving antihypertensive medication, and had an established diagnosis of chronic hypertension. In addition, subjects had at least one other CVD risk factor including diabetes, dyslipidemia, obesity, chronic kidney disease, microalbuminuria, current smoking, or age >55 for men or >65 for women. All participants provided written informed consent in order to participate in the study. Patients were evaluated consecutively during the enrollment period and excluded if they had pre-existing CVD defined as myocardial infarction, prior percutaneous or surgical coronary revascularization, stroke, cerebrovascular revascularization, or peripheral arterial disease. Additional exclusions were valvular heart disease, end-stage renal disease, inflammatory disease, active substance abuse, or projected life expectancy <12 months. Non-invasive vascular structure and function markers as well as inflammatory biomarkers were obtained on a single day. The Colorado Multiple Institutional Review Board approved the study protocol.

### Inflammatory markers

A venous plasma sample was obtained from all subjects. Patients were asked to fast and refrain from smoking from the night before their appointment. We chose two markers of systemic inflammation, high sensitivity C-reactive protein (hsCRP) because it has been studied extensively as a risk factor^5 ^and lipoprotein-associated phospholipase A2 (Lp-PLA2) given its purported specificity for inflammation localized to atherosclerotic plaque including carotid arteries [10-13]. Levels of Lp-PLA2 mass and activity were measured (PLAC; diaDexus Inc, South San Francisco, CA) by a dual monoclonal antibody immunoassay.^13 ^Samples were assayed for hsCRP with a high-sensitivity assay (hsCRP Flex^®^, Dimension Vista). This assay was validated against the Roche Hitachi Modular assay and found to have acceptable precision and reproducibility [14].

### Vascular function measures

All measurements were performed in a quiet room with controlled ambient temperature. Blood pressure was measured in duplicate in the supine position using the non-dominant arm in all subjects. Brachial-ankle pulse wave velocity (PWV) was derived from the pulse transit time between and the estimated path length between proximal and distal arterial sites expressed as cm/sec. Applanation tonometry (HEM-9000AI Omron Healthcare, Bannockburn, Illinois) was performed at the radial artery to derive the augmentation index (AIx) and central aortic pressure (cAP). AIx was calculated as the difference between the first (ejection) and second (reflected) peaks of the arterial waveform, expressed as a percentage of the pulse pressure, where higher AIx values reflect greater vascular stiffness.

One ultrasonographer assessed flow-mediated dilation (FMD) of the brachial artery in the non-dominant arm, which was immobilized and scanned above the antecubital fossa using a 5-12 MHz linear array transducer (Phillips Medical Systems model iE33, Bothell, WA). Arterial images were obtained before (baseline) and after (maximal) inflation of a cuff at pressures >40 mm Hg above systolic blood pressure for five minutes. Longitudinal images were scanned and captured (in mm) at end diastole. FMD was defined as [maximal - baseline brachial artery diameter] divided by baseline brachial artery diameter × 100 and expressed as percentages.

### Carotid intima-media thickness

Measures of maximal carotid IMT were obtained in the supine position by a single ultrasonographer. Longitudinal B-mode ultrasound images were obtained among subjects with the head turned 45 degrees from the area scanned. Gain settings were optimized to acquire far wall arterial images and limit echogenicity of the lumen. A linear array probe (Phillips Sonos 5500, Netherlands) was used for all image acquisition. The sonographer obtained 3 longitudinal views of both internal carotid arteries for a total of 6 IMT images per subject as previously described [15]. The internal carotid artery was defined to include the bulb and the initial 10 mm of vessel distal to separation of external from internal arteries. High resolution images were stored digitally, and read off-line by trained interpreters blinded to clinical characteristics of study participants. Near and fall wall thickness were calculated as the maximum distance between the lines.

### Statistical Analysis

Mean and standard deviations for all normally distributed variables were calculated. Pulse wave velocity was normalized after transformation by the relationship P-inverse = - (1/PWV) - 0.0006)/0.00011) [16]. Carotid IMT was the primary outcome variable. Pearson correlation coefficients or Student's t-test were used to assess univariate relationships between carotid IMT and candidate independent variables. Multivariate linear regression models were used to assess the association between carotid IMT and the inflammatory, vascular stiffness, and endothelial function variables after adjusting for variables used in the Framingham general risk profile including age, gender, systolic blood pressure, anti-hypertensive medication use, total and HDL-cholesterol, diabetes, and smoking status. Because all subjects were receiving anti-hypertensive medications, this variable was not considered further. P-values < 0.05 were considered statistically significant. Receiver operator characteristic curves to predict high carotid IMT as a dichotomous variable were also constructed for Framingham risk score, PWV, and AIx. We tested interactions with each independent variable and ethnicity in another set of models to test the hypothesis that the relationship between the outcome variables and the independent variables would be similar between Latino and non-Latino white subgroups. Following data quality assurance measures, SAS Version 8.0 (Cary, NC) was used for all statistical analyses.

## Results

**Figure 1:**
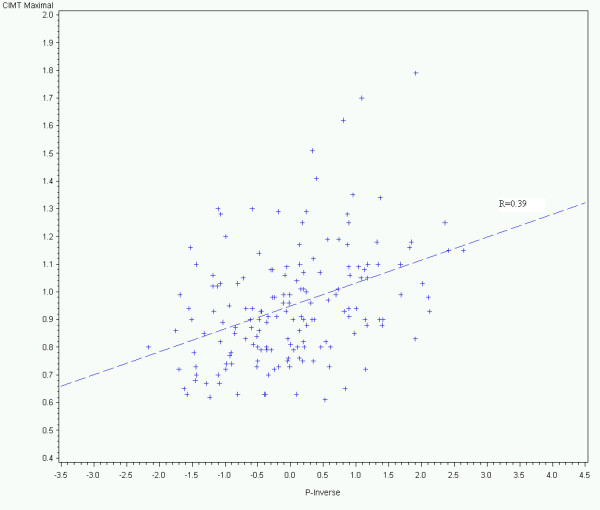
**Relationship of pulse wave velocity to carotid intima media thickness**. The relationship between the inverse of the brachial-ankle pulse wave velocity (PWV) in cm/second and the carotid intima media thickness in mm. P-inverse is - (1/PWV) - 0.0006)/0.00011). The resulting Z (P-inverse) quantity has a mean of 0 and an SD of 1. The negative sign is used to ensure that correlations with PWV retain the same direction when compared to P-inverse.

Baseline characteristics of the sample are shown in Table 1. The mean age was 62 years, two thirds were female and two-thirds were Latino. Nearly half had diabetes. Consistent with a sample drawn from a safety net institution, nearly half had not completed high school and over 80% were unemployed or disabled. Mean LpPLA2 was 173 ± 59 ng/ml. In contrast with hsCRP, only 21% of subjects would be classified as high-risk, using the LpPLA2 cut-point of >200 ng/ml [17]. Mean hsCRP was 5.25 ± 6.65 mg/L and over 63% of the cohort would be classified as high-risk using the recently established cut-point of 2 mg/L used in the JUPITER trial [18]. Univariate relationships between independent variables and carotid IMT are shown in Table 2. The inflammatory, and vascular function variables associated with cIMT at p < 0.25 on univariate analysis were PWV, AIx, cAP and LpPLA_2 _activity rank. PWV had the strongest linear relationship (r = + 0.39, p < 0.0001) with carotid IMT (Figure 1). For high carotid IMT (>0.8 mm) C-statistics assessing discrimination for PWV, AIx and Framingham risk score (adjusting for age, gender, systolic blood pressure, and ethnicity) were 0.72, 0.63, and 0.51 respectively. Results of the multiple linear regression analyses with standardised coefficients are shown in Table 3. After adjusting for Framingham risk factors, AIx, cAP, and LpPLA2 activity were no longer significantly associated with carotid IMT (all p > .10). Only PWV remained associated with carotid IMT independent of the Framingham general risk profile parameters (p = .016). Additional analyses indicated no statistically significant interactions between the Framingham risk factors and other independent variables and ethnicity (all p > .05) when predicting carotid IMT. For the interaction between PWV and ethnicity when predicting carotid IMT, the p-value was 0.518.

**Table 1:** Patient Characteristics (n = 177)

Age, years	61.5 (9.7)
Female gender	66.7%
Ethnicity	
Hispanic	66.7%
Non-Hispanic White	33.3%
Education	
College graduate	8.5%
Higher education	19.3%
High school graduate	19.8%
Some high school	21.5%
No high school	31.1%
Employment Status	
Employed	19.2%
Unemployed	46.9%
Disabled	33.9%
Body mass index, kg/m^2^	33.0 (6.7)
Blood pressure, mmHg	142.2 ± 22.1/81.7 ± 12.3
Total cholesterol, mg/dl	181.5 (39.6)
Triglycerides, mg/dl	170.9 (105.2)
HDL cholesterol, mg/d	51.0 (12.8)
LDL-cholesterol, mg/dl	96.8 (33.3)
Diabetes Mellitus	49.2%
Current Smoker	22.0%
Framingham risk score*	11.6 (8.0), 1-53
LpPLA2^† ^mass, ng/mL	173.5 (59.0)
LpPLA2 activity, nmol/min/mL	134.8 (34.3)
Central aortic pressure, mm Hg	59.5 (18.1)
Augmentation index	86.3% (12.7)
Pulse wave velocity, cm/sec	1723.3 (349)
Flow-mediated dilation	10.5% (8.1)
Carotid intima-media thickness, mm	0.95 (0.22)
Use of Hypertension Medication	90.4%
Use of Hydroxy-3-methylglutaryl Co-enzyme A reductase inhibitors (statins)	59.9%

Values expressed as mean + standard deviation (SD) or as percentages (%). hsCRP = high sensitivity C-reactive protein. *mean (SD) and range^† ^LpPLA2 = Lipoprotein-associated phospholipase A2.

**Table 2:** Univariate relationships with Carotid Intima Media Thickness

N = 177	Carotid intima-media thickness	
	**Correlation coefficient**	**P**

Age (years)	0.369	<.0001
Systolic blood pressure (mm Hg)	0.207	0.006
Total cholesterol (mg/dL)	0.054	0.476
HDL cholesterol (mg/dL)	0.096	0.210
Pulse wave velocity (cm/s)	0.389	<.0001
Augmentation Index (%)	0.123	0.130
Central aortic pressure (mm Hg)	0.254	0.005
hsCRP (mg/L)	-0.004	0.959
LPPLA2 Mass rank	0.034	0.653
LPPLA2 Activity rank	0.110	0.149
Flow-mediated dilation (%)	-0.087	0.270
Gender	-0.170	0.007
Current Smoker	0.023	0.713
Diabetes	-0.046	0.461

hsCRP = high sensitivity C-reactive protein. LpPLA2 = Lipoprotein-associated phospholipase A2. HDL = high density lipoprotein

**Table 3:** Multivariate relationships with carotid intima-media thickness (n = 177)

	Framingham factors alone		Framingham factors + PWV		Framingham factors + AIx		Framingham factors + cAP		**Framingham factors + LpPLA**_**2**_	
	
	estimate	P	estimate	P	estimate	P	estimate	P	estimate	P
Age	0.074	**<.0001**	0.045	**0.016**	0.073	**<.0001**	0.068	**0.004**	0.075	**<.0001**
Female Gender	-0.111	**0.002**	-0.113	**0.001**	-0.127	**0.002**	-0.144	**0.001**	-0.113	**0.002**
Systolic BP	0.025	0.119	0.017	0.323	0.012	0.507	0.008	0.819	0.025	0.115
Total Cholesterol	0.014	0.397	0.003	0.862	0.005	0.772	0.005	0.809	0.018	0.296
HDL Cholesterol	0.018	0.303	0.022	0.205	0.024	0.201	0.024	0.245	0.017	0.322
Current Smoker	0.011	0.782	-0.003	0.937	0.023	0.596	0.007	0.893	0.018	0.659
Diabetes	-0.008	0.796	-0.010	0.745	0.000	0.994	-0.025	0.562	-0.010	0.763

Pulse wave velocity			0.049	**0.016**						
Augmentation Index					0.021	0.295				
Central Aortic Pressure							0.034	0.384		
LpPLA_2_									-0.013	0.441

## Discussion

In this cross-sectional study of a mixed-ethnicity population, PWV, a measure of arterial stiffness, was associated with preclinical carotid atherosclerosis independent of Framingham risk factors. To our knowledge, this is the first study to demonstrate that PWV is associated with preclinical atherosclerosis among a Latino-predominant population. Findings were similar in both Latino and non-Latino White hypertensive subjects, suggesting that measures of arterial stiffness could play a role in CVD risk stratification within the growing US safety net population. Moreover, two other arterial stiffness measures, two inflammatory markers, and a traditional measure of endothelial function were not associated with carotid intima media thickness.

Overall, most studies to date included patients with a wide range of ages and underlying conditions but a limited range of race and ethnicity. In particular, none included substantial numbers of Latinos. Moreover, very few studies have compared the relative value of arterial stiffness, inflammatory markers, and endothelial function as correlates of CVD beyond isolated coronary disease events. An epidemiological study of 2,191 elderly (age at baseline 70-79) African American and White subjects, assessed the predictive value of PWV, ankle brachial index (ABI), and inflammatory markers (hsCRP, interleukin-6, and TNF-α) to Framingham risk factors [19]. The outcome variable was limited to coronary disease events. PWV, ABI, and interleukin-6 were independent predictors, and the model with the best discriminative capacity added both ABI and interleukin-6 to Framingham factors. These results differ from our finding that PWV was associated with preclinical cardiovascular disease whereas inflammatory markers were not. By contrast, a study of 558 patients with hypertension and without baseline CVD, added PWV and FMD in Cox multi-variable models that had occurrence of CVD events including stroke as the outcome variable [20]. PWV was related to the occurrence of CVD events while FMD was not, consistent with the results of the current study in a similar hypertensive cohort. Our findings are also consistent with the current consensus opinion that PWV is the gold standard method for assessing arterial stiffness [21].

Our results extend to a multi-ethnic population findings of an association between arterial stiffness and cardiovascular outcomes. In a recent meta-analysis, Vlachopoulos et al [22] combined results of 17 longitudinal studies and found a relative risk of 1.47 for cardiovascular events associated with each standard deviation increase in PWV. These 17 studies varied in the degree to which they accounted for Framingham risk factors and in the degree to which they considered a full range of non-coronary CVD outcomes. The most comprehensive study was performed by Mitchell et al [16], and was consistent with our study in that PWV, but not AIx or cAP, was a significant predictor of CVD events independent of the Framingham general risk profile parameters.

A number of factors should be considered in interpreting the results of the current investigation. This was a cross-sectional study and was not powered to predict CVD events. With a larger sample size, additional vascular function variables could have reached statistical significance. Relative to PWV, however, cAP and AIx are less direct measures of arterial stiffness, more sensitive to differences in heart rate and body size, and may be more technically challenging to measure thereby limiting their utility in risk stratification. The suggestion that LpPLA_2 _may have greater predictive value than hsCRP in high-risk populations deserves further exploration since hsCRP values were uniformly elevated in this safety net population. The absence of even a univariate association between hsCRP and carotid IMT in the current study is informative given the widespread use of this marker in clinical practice. A recent guideline affirms that measurement of hsCRP is not recommended for risk stratification in asymptomatic higher-risk adults, nor among older low-risk adults [23]. Confirmatory longitudinal studies comparing vascular function and inflammatory markers as predictors of CVD in samples with mixed race and ethnicity are needed to better assess the strength and clinical implications of this association. In addition, the prognostic significance of brachial-ankle PWV is less established compared with carotid-femoral PWV. However, brachial-ankle PWV, is more easily obtained and has been shown to be highly correlated (r = 0.76, p < 0.0001) with carotid-femoral PWV values among hypertensive patients [24]. Because of its strong relationship with aortic pulse wave velocity, brachial-ankle PWV measurements offers both practical and theoretic advantages over the more frequently used carotid-femoral method [25], though confirmatory studies with carotid-femoral PWV are warranted. One strength of the current analysis is its focus on a traditionally under-studied safety net population. Evaluating sociodemographically vulnerable subjects serves to broaden the applicability of research findings to those individuals typically excluded from research trials; in our study, the majority of participants were women and Latinos in marked contrast to typical cardiovascular trials [26].

## Conclusions

Despite its strong association with cardiovascular mortality among hypertensive patients [27] and endorsement as a gold standard for vascular stiffness, the value of PWV as a therapeutic target remains uncertain. Nonetheless, our study demonstrates that PWV, in contrast to inflammatory biomarkers, has predictive value for preclinical atherosclerosis beyond the Framingham risk profile among subjects of mixed ethnicity. Given ongoing concerns about the inaccuracy of the Framingham risk score in racial/ethnic minorities [28] and the importance of considering overall CVD risk in such individuals, PWV warrants further evaluation in both epidemiologic and interventional studies.

## Competing interests

The authors declare that they have no competing interests.

## Authors' contributions

MJK was the lead on non-invasive hemodynamic data and carried out primary drafting of the paper and study design, CSL led the biomarker analysis and contributed to study design and substantive edits, PH performed all statistical analyses, tables' and figures and was involved in quality control. EK led the acquisition and interpretation of data and made substantive intellectual contributions. MD was the senior statistician and provided critical content review. ROE made substantial contributions to conception and design. FAM was responsible for IMT measurements and interpretation as well as key analysis. EPH conceived of and designed the overall study and contributed substantial time to analysis and framing the paper. All authors read and approved the final manuscript.

## Pre-publication history

The pre-publication history for this paper can be accessed here:

http://www.biomedcentral.com/1471-2261/11/15/prepub

## References

[B1] D'Agostino RB Sr Vasan RS Pencina MJ Wolf PA Cobain M Massaro JM Kannel WB General cardiovascular risk profile for use in primary care: the Framingham Heart Study Circulation 2008 117 743 753 18212285 10.1161/CIRCULATIONAHA.107.699579

[B2] Wilson PW D'Agostino RB Sr Levy D Belanger AM Silbershatz H Kannel WB Prediction of coronary heart disease using risk factor categories Circulation 1998 97 1837 1847 9603539 10.1161/01.cir.97.18.1837

[B3] Marma AK Ning H Lloyd-Jones DM Impact of a change to global cardiovascular risk estimation in lipid-lowering guidelines: Finding from the National Health and Nutrition Examination Survey (NHANES) 2001-2006 Circulation 2009 120 S424 10.1161/CIRCULATIONAHA.108.835470

[B4] Lloyd-Jones D Adams RJ Brown TM on behalf of the American Heart Association Statistics Committee and Stroke Statistics Subcommittee Heart disease and stroke statistics--2010 update: a report from the American Heart Association Circulation 2010 121 e1 e170 10.1161/CIRCULATIONAHA.109.878314 20048228

[B5] Ridker PM Buring JE Rifai N Cook NR Development and validation of improved algorithms for the assessment of global cardiovascular risk in women: the Reynolds Risk Score JAMA 2007 297 611 619 10.1001/jama.297.6.611 17299196

[B6] Heffernan KS Fahs CA Iwamoto GA Jae SY Wilund KR Woods JA Fernhall B Resistance exercise training reduces central blood pressure and improves microvascular function in Afrincan American and white men Atherosclerosis 2009 207 220 226 10.1016/j.atherosclerosis.2009.03.043 19410255

[B7] http://www.censusscope.org/us/map_hispanicpop.html Accessed 25 December 2009

[B8] Hanratty R Estacio RO Dickinson LM Chandramouli V Steiner JF Havranek EP Latino Using Cardio Health Actions to Reduce Risk study investigators Testing electronic algorithms to create disease registries in a safety net system J Health Care Poor Underserved 2008 19 452 465 10.1353/hpu.0.0027 18469416 PMC2561200

[B9] Gabow P Eisert S Wright R Denver Health: a model for the integration of a public hospital and community health centers Ann Int Med 2003 138 143 149 12529097 10.7326/0003-4819-138-2-200301210-00016

[B10] Lavi S McConnell JP Rihal CS Prasad A Mathew V Lerman LO Lerman A Local production of lipoprotein-associated phospholipase A2 and lysophosphatidylcholine in the coronary circulation: association with early coronary atherosclerosis and endothelial dysfunction in humans Circulation 2007 115 2715 2721 10.1161/CIRCULATIONAHA.106.671420 17502572

[B11] Kolodgie FD Burke AP Skorija KS Ladich E Kutys R Makuria AT Virmani R Lipoprotein-associated phospholipase A2 protein expression in the natural progression of human coronary atherosclerosis Arterioscler Thromb Vasc Biol 2006 26 2523 2529 10.1161/01.ATV.0000244681.72738.bc 16960105

[B12] Mannheim D Herrmann J Versari D Gossl M Meyer FB McConnell JP Enhanced expression of Lp-PLA2 and lysophosphatidylcholine in symptomatic carotid atherosclerotic plaque Stroke 2008 39 1448 1455 10.1161/STROKEAHA.107.503193 18356547 PMC4360896

[B13] Davidson MH Corson MA Alberts MJ Anderson JL Gorelick PB Jones PH Consensus panel recommendation for incorporating lipoprotein-associated phospholipase A2 testing into cardiovascular disease risk assessment guidelines Am J Cardiol 2008 101 51F 57F 10.1016/j.amjcard.2008.04.019 18549872

[B14] Clinical and Laboratory Standards Institute (CLSI) Methods comparison and bias estimation using patient samples; approved guideline-second edition CLSI document EP9-A2 (ISBN 1-56238-472-4) 2002 CLSI, 940 West Valley Road, Suite 1400, Wayne PA 19087-1898 USA

[B15] O'Leary DH Polak JF Wolfson SK Jr Use of sonography to evaluate carotid atherosclerosis in the elderly. The Cardiovascular Health Study. CHS Collaborative research Group Stroke 1991 22 1155 63 1926258 10.1161/01.str.22.9.1155

[B16] Mitchell GF Hwang SJ Vasan RS Larson MG Pencina MJ Hamburg NM Arterial stiffness and cardiovascular events: the Framingham Heart Study Circulation 2010 121 505 511 10.1161/CIRCULATIONAHA.109.886655 20083680 PMC2836717

[B17] Kardys I Oei HHS van der Meer IM Horman A Breteler MMB Witteman JCM Lipoprotein-associated phospholipase A2 and measures of extracoronary atherosclerosis: the Rotterdam study Aterioscler Thromb Vasc Biol 2006 26 631 636 10.1161/01.ATV.0000201289.83256.cf 16373603

[B18] Ridker PM Danielson E Fonseca FA Genest J Gotto AM Jr Kastelein JJ JUPITER Study Group Rosuvastatin to prevent vascular events in men and women with elevated C-reactive protein N Engl J Med 2008 359 2195 2207 10.1056/NEJMoa0807646 18997196

[B19] Rodondi N Marques-Vidal P Butler J Sutton-Tyrrel K Comuz J Satterfield S Harris T Bauer DC Ferrucci L Vittinghoff E Newman AB Markers of atherosclerosis and inflammation for prediction of coronary heart disease in older adults Am J Epidemiol 2010 171 540 549 10.1093/aje/kwp428 20110287 PMC2842214

[B20] Terai M Ohishi M Ito N Takagi T Tatara Y Kaibe M Comparison of arterial functional evaluations a a predictor cardiovascular events in hypertensive patients: the Non-invasive Atherosclerotic Evaluation in Hypertension (NOAH) study Hypertens Res 2008 31 1135 1145 10.1291/hypres.31.1135 18716361

[B21] Laurent S Cockcroft J Van Bortel L Boutouyrie P Giannattasio C Havoz D Expert consensus document on arterial stiffness: methodological issues and clinical applications Eur Heart J 2006 27 2588 2605 10.1093/eurheartj/ehl254 17000623

[B22] Vlachopoulos C Aznaouridis K Stefanidis C Prediction of cardiovascular events and all-cause mortality with arterial stiffness: A systematic review and meta-analysis J Am Coll Cardiol 2010 55 1318 1327 10.1016/j.jacc.2009.10.061 20338492

[B23] Greenland P Alpert JS Beller GA Benjamin EJ Budoff MJ Fayad ZA Foster E Hlatky MA Hodgson JMcB Kushner FG Lauer MS Shaw LJ Smith SC Jr Taylor AJ Weintraub WS Wenger NK 2010 ACCF/AHA guideline for assessment of cardiovascular risk in asymptomatic adults: executive summary: a report of the American College of Cardiology Foundation/American Heart Association Task Force on Practice Guidelines J Am Coll Cardiol 2010 56 2182 99 10.1016/j.jacc.2010.09.002 21144964

[B24] Munakata M Ito N Nunokawa T Yoshinaga K Utility of automated brachial ankle pulse wave velocity measurements in hypertensive patients Am J Hypertens 2003 16 653 657 10.1016/S0895-7061(03)00918-X 12878371

[B25] Sugawara J Hayashi K Yokio T Cortez-Cooper MY DeVan AE Anton MA Tanaka H Brachial-ankle pulse wave velocity: an index of central arterial stiffness? J Hum Hypertens 2005 19 401 406 10.1038/sj.jhh.1001838 15729378

[B26] Hoel AW Kayssi A Brahmanandam S Belkin M Conte MS Nguyen LL Under-representation of women and ethnic minorities in vascular surgery randomized controlled trials J Vasc Surg 2009 50 349 54 10.1016/j.jvs.2009.01.012 19631869 PMC2759770

[B27] Laurent S Boutouyre P Asmar R Gautier I Laloux B Guize L Aortic stiffness is an independent predictor of all-cause and cardiovascular disease mortality in hypertensive patients Hypertension 2001 37 1236 1241 11358934 10.1161/01.hyp.37.5.1236

[B28] D'Agostion RB Sr Grundy S Sullivan LM Wilson P CHD Risk Prediction Group Validation of the Framingham coronary heart disease risk prediction scores. Results of a multiple ethnic groups investigation JAMA 2001 286 180 187 11448281 10.1001/jama.286.2.180

